# Evaluating Gene Expression in C57BL/6J and DBA/2J Mouse Striatum Using RNA-Seq and Microarrays

**DOI:** 10.1371/journal.pone.0017820

**Published:** 2011-03-24

**Authors:** Daniel Bottomly, Nicole A. R. Walter, Jessica Ezzell Hunter, Priscila Darakjian, Sunita Kawane, Kari J. Buck, Robert P. Searles, Michael Mooney, Shannon K. McWeeney, Robert Hitzemann

**Affiliations:** 1 Research Service, Veterans Affairs Medical Center, Portland, Oregon, United States of America; 2 Oregon Clinical and Translational Research Institute, Oregon Health & Science University, Portland, Oregon, United States of America; 3 Department of Behavioral Neuroscience, Oregon Health & Science University, Portland, Oregon, United States of America; 4 Massively Parallel Sequencing Shared Resource, Oregon Health & Science University, Portland, Oregon, United States of America; 5 Division of Bioinformatics and Computational Biology, Medical Informatics & Clinical Epidemiology, Oregon Health & Science University, Portland, Oregon, United States of America; 6 Division of Biostatistics, Public Health & Preventative Medicine, Oregon Health & Science University, Portland, Oregon, United States of America; 7 OHSU Knight Cancer Institute, Oregon Health and Science University, Portland, Oregon, United States of America; University of Chicago, United States of America

## Abstract

C57BL/6J (B6) and DBA/2J (D2) are two of the most commonly used inbred mouse strains in neuroscience research. However, the only currently available mouse genome is based entirely on the B6 strain sequence. Subsequently, oligonucleotide microarray probes are based solely on this B6 reference sequence, making their application for gene expression profiling comparisons across mouse strains dubious due to their allelic sequence differences, including single nucleotide polymorphisms (SNPs). The emergence of next-generation sequencing (NGS) and the RNA-Seq application provides a clear alternative to oligonucleotide arrays for detecting differential gene expression without the problems inherent to hybridization-based technologies. Using RNA-Seq, an average of 22 million short sequencing reads were generated per sample for 21 samples (10 B6 and 11 D2), and these reads were aligned to the mouse reference genome, allowing 16,183 Ensembl genes to be queried in striatum for both strains. To determine differential expression, ‘digital mRNA counting’ is applied based on reads that map to exons. The current study compares RNA-Seq (Illumina GA IIx) with two microarray platforms (Illumina MouseRef-8 v2.0 and Affymetrix MOE 430 2.0) to detect differential striatal gene expression between the B6 and D2 inbred mouse strains. We show that by using stringent data processing requirements differential expression as determined by RNA-Seq is concordant with both the Affymetrix and Illumina platforms in more instances than it is concordant with only a single platform, and that instances of discordance with respect to direction of fold change were rare. Finally, we show that additional information is gained from RNA-Seq compared to hybridization-based techniques as RNA-Seq detects more genes than either microarray platform. The majority of genes differentially expressed in RNA-Seq were only detected as present in RNA-Seq, which is important for studies with smaller effect sizes where the sensitivity of hybridization-based techniques could bias interpretation.

## Introduction

Sandberg et al. [1] appear to have been the first to use microarrays to examine differential brain gene expression between two inbred mouse strains (C57BL/6 [B6] and 129SvEv). Using a 1.8 fold-change threshold, these authors found that ∼1% of the transcripts detected as present were differentially expressed. Subsequent studies have examined differential brain gene expression within large panels of inbred mouse or rat strains [2], [3], [4], [5] and in segregating rodent populations [6], [7], [8]. Recombinant inbred strains and F_2_ populations have been used to generate expression QTL (eQTL) maps which in turn are often integrated with behavioral quantitative trait loci (bQTL) maps [4], [5], [9]. Improvements in both microarray technology and analytical techniques made it possible to measure changes in brain gene expression quite accurately; importantly the cumulative data record has indicated that most differences in expression between inbred strains or populations derived from inbred strains, such as selectively bred lines [10], are actually quite small (15 to 30%). To some extent the small changes reflect the fact that the hybridization isotherms for oligonucleotide arrays are frequently not linear due to probe saturation [11]. This effect may well be accentuated in brain because of the high degree of alternative splicing [12]. A second problem with oligonucleotide arrays and one that is specific to inbred strains or populations derived from inbred strains is the effect of SNPs [8], [13]. Rodent oligonucleotide arrays are based upon the sequence of the B6 mouse or Brown-Norway (BN) rat strains. Even inbred strains closely related to the B6 or BN may differ by several million SNPs [14], which in turn can cause significant hybridization artifacts [13]. Masking for SNPs can improve this situation but in some cases results in deleting a probe (Illumina) or a probeset (Affymetrix) from the analysis. A third problem with oligonucleotide arrays are the annotation and summarization issues associated with predefined reporters/probes. Finally, because microarrays primarily interrogate the 3′ untranslated region (3′-UTR), they provide relatively little information about alternative splicing. The Affymetrix 1.0 Exon ST array collects data on alternative splicing but when used to detect differential alternative splicing is particularly sensitive to the “SNP effect” due to the smaller number of probes per probeset.

The emergence of next-generation sequencing (NGS) and the RNA-seq application provides a clear alternative to oligonucleotide arrays for detecting differential gene expression and effectively deals with the problems noted above. Mortazavi et al. [15] compared mouse liver gene expression (B6 strain only) between data generated by the short-read Illumina/Solexa 1G platform and data generated from the Affymetrix 430 2.0 array. The short-read metric of comparison was reads per kilobase of exon model per million mapped reads (RPKM). At the gene level, the two platforms agreed reasonably well. However, RNA-Seq also generated new data. While 90% of the uniquely mapped reads fell within known exons, the additional data suggested “new and revised gene models, including changed or additional promoters, exons and 3′-UTRs as well as new candidate microRNA precursors.” Mortazavi et al. [15] were also able to detect >100,000 splice sites and noted that ∼3,500 genes expressed one or more alternate internal splices. Subsequent studies in mouse and other species, e.g. yeast, have confirmed that RNA-seq and microarrays yield similar data at the level of gene expression [16], [17], [18]. There have been to our knowledge no studies that have used RNA-seq to examine differential expression across inbred strains. However, with a similar application in mind, Bullard et al. [19] examined the various normalization paradigms that are optimal to detect differential gene expression; they concluded that upper quartile normalization introduces the least amount of bias.

The current study compares RNA-Seq with two microarray platforms (Affymetrix MOE 430 2.0 and Illumina MouseRef-8 v2.0) to detect differential striatal gene expression between the B6 and DBA/2J (D2) inbred mouse strains. There are several reasons for comparing the B6 and D2 strains. First, the B6 is the reference strain for the mouse genome and the D2 strain is one of several strains that are being completely sequenced as part of the Sanger Mouse Genome project. Also there are extensive gene expression datasets comparing the B6 and D2 strains and both recombinant inbreds and F_2_ intercrosses derived from these strains; included in these datasets are data from the striatum [8], [20], [21]. Finally, there is extensive phenotypic data comparing the B6 and D2 strains [see 22] including data for behavioral, anatomical and behavioral striatal-related-phenotypes [2], [23], [24]. We show that by using stringent data processing requirements differential expression as determined by RNA-Seq is concordant with both the Affymetrix and Illumina platforms in more instances than it is concordant with only one, and that instances of conflicting directions of fold change are rare. The large dynamic range of RNA-Seq detects thousands more genes than seen on the microarrays. The additional information gained by using this technology illustrates the value of RNA-Seq.

## Methods

### RNA preparation

Naïve, adult, male B6 and D2 strain mice were used in the RNA-Seq and Illumina microarray experiment while both genders were utilized in the Affymetrix microarray experiment. This animal study was reviewed and approved by the Portland Veterans Affairs Medical Center Institutional Animal Care and Use Committee under protocol ID VA1509. Animals were sacrificed by cervical dislocation and the brains removed. The striatum was dissected as follows. A 1.5-mm coronal slice of tissue was removed for which the caudal boundary was the optic chiasm. The slice was laid rostral surface down on an ice-cold Petri dish, and the material surrounding the striatum was carefully removed. From the caudal to rostral surface, the striatum decreases in size and thus an angled cut was used to remove additional non-striatal material. After dissection, the tissue was either flash frozen in liquid nitrogen or stored in RNAlater (Qiagen, Valencia CA). Total RNA was isolated using TRIzol® reagent (Invitrogen, Carlsbad CA) using a one-step guanidine isothiocyanate procedure. RNA samples were evaluated by ultraviolet spectroscopy for purity and concentration (NanoDrop, Wilmington, DE) and were assessed further for RNA integrity on the Agilent Bioanalyzer (Santa Clara, CA). All samples had an RNA Integrity Number (RIN) of 8 or better.

### RNA-seq

Total RNA from 21 male mice (10 B6 and 11 D2) was provided to the Oregon Health & Science University Massively Parallel Sequencing Shared Resource facility [25] for transcriptome sequencing (NCBI SRA accession number: SRA026846.1). Libraries were prepared using the Illumina mRNA-Seq Sample Preparation Kit (San Diego, CA). Briefly, poly(A)+ RNA was recovered from 1 µg of total RNA using two rounds of isolation with oligo-dT - coated Sera-Mag magnetic beads. The recovered poly(A)+ RNA was then chemically fragmented. RNA fragments were converted to cDNA using SuperScript II and random primers. The second strand was synthesized using RNaseH and DNA Pol I. The ends of the cDNA were repaired using T4 DNA polymerase, T4 polynucleotide kinase, and Klenow DNA polymerase. A single adenosine was added to the 3′ end using Klenow fragment (3′ to 5′ exo minus). Adaptors were attached to the ends of the cDNA using T4 DNA ligase. 300 bp fragments were extracted from a 2% low range ultra agarose sizing gel. The 300 bp fragment was then amplified by 15 cycles of polymerase chain reaction using (PCR) Phusion DNA polymerase. Libraries were validated with an Agilent Bioanalyzer (Santa Clara, CA). Libraries were diluted to 10 pM and applied to an Illumina flow cell using the Illumina Cluster Station. Sequencing was performed on an Illumina GAIIx. Sequences were 76 cycle single read except for the third flowcell, which was 70 cycles. The resulting data were processed using the Illumina CASAVA package as described in version 1.6 of the CASAVA user guide.

All reads were realigned to the NCBI m37 version of the mouse genome assembly using the Bowtie [26] short read alignment program considering the 22 chromosome assemblies. Each read was trimmed to a length of 43 bases and a seeded alignment was carried out using the first 32 bases allowing up to 2 mismatches. Only uniquely mapping reads were used in this analysis. Exon start and stop locations for NCBI m37 assembly were downloaded from Ensembl [27], and for each gene, the union exons were created as follows. For each Ensembl gene, annotated exons were merged to create a set of non-redundant exons for a particular gene. For example, if one annotated transcript had an exon that overlapped exon boundaries in a second annotated transcript for a given gene, the union exon boundaries would consist of the start location of the first exon and the stop location of the second exon. Because we did not perform strand specific RNA-Seq, if annotated exons overlapped between genes, these intervals were removed from the overall gene expression calculations similar to the union intersection exons recently proposed [19]. A read was counted using the Genominator [19] package from Bioconductor 2.6 if the start position was located in one of our determined union exons. A gene-level representation was then created by summing the counts for each union exon attributed solely to a given gene. Genes with read counts of zero in all 21 lanes were removed, as well as genes that contained a zero read count in at least one B6 and one D2 sample. Total count normalization [19] and the fitting of the Poisson model was performed in R [28], and upper quartile normalization along with the negative binomial exact test was performed using the edgeR Bioconductor package. The genes with the log transformed average read count as determined by edgeR of less than −20 were flagged as being low read count in **Dataset S1**. The single nucleotide (SNP) correction of the NCBI m37 genome assembly was performed using a custom perl script using SNPs obtained for the D2 strain from the Sanger Mouse Genomes Project [14]. To create a synthetic D2 sequence, for each SNP the D2 allele was inserted in the reference (B6) sequence at the specified location. As the reference genome was repeat masked, if an ‘N’ was present at the reference position, the D2 base was not inserted but left as an ‘N’. All boxplots were generated using the ggplot2 R package [29].

### Microarray

High quality samples containing 2 µg of total RNA were sent to the Oregon Health & Science University Gene Microarray Shared Resource facility for labeling and hybridization on microarray chips. The procedures used precisely followed Affymetrix's or Illumina's specifications, respectively [30]. All microarray data are MIAME compliant and the raw data has been deposited in GEO with accession number GSE26024.

An independent group of 20 mouse samples (5 male D2, 5 female D2, 5 male B6, and 5 female B6 were assessed using the Affymetrix MOE 430 2.0 array. These data were analyzed using the Robust Multichip Average methodology [31] on perfect match probes with the proposed background correction, quantile normalization, and summarization procedures as in previous work [13], [32], [33]. Differential expression was determined using the limma Bioconductor [34] package by fitting a linear model also incorporating gender status. Individual probes that spanned a SNP between the B6 and D2 strains and probes with non-unique alignments were masked [13]. For the purposes of comparing across platforms, we used the Ensembl mappings of microarray probesets [27]. If a probeset did not map within a unique exon of an Ensembl build 59 gene, it was excluded from these analyses. Any probeset that had fewer than four probes remaining (of the 11 in each probeset) after masking was excluded from our analyses. To remove unreliable calls and set a threshold for the Affymetrix probesets, we used default settings with the MAS5 method in the affy package of Bioconductor. The MAS5 detection call flags the probeset as ‘Present’ or detected (p<0.04), ‘Marginal’ (0.04<p<0.06) or ‘Absent’ or undetected (p>0.06). Probesets with an ‘Absent’ call in at least one B6 and one D2 sample were excluded. All differential expression p-values were false discovery rate (FDR) adjusted using the q-value bioconductor package [35]. Fold change was computed using average anti-log RMA values for each strain.

A total of 24 male mice (12 D2 and 12 B6) were assessed using the Illumina MouseRef-8 v2.0 array. These include an independent group of 5 D2 and 5 B6 mice, and a group of 7 B6 and 7 D2 samples that were also analyzed using RNA-seq. The Illumina array data were analyzed with the lumi Bioconductor package within R [36]. The data were transformed using the variance stabilization transformation (VST) [37] and normalized using the robust spline normalization (RSN) [36]. The threshold for a probe to detect expression as ‘present’ was set at p<0.05 as determined by lumi. Probes were excluded from our analyses if they did not map perfectly to a single genomic location on the NCBI m37 assembly of the mouse genome. Similar to the methods for Affymetrix, we used Ensembl mappings of Illumina probes for comparisons across platforms, so probes were excluded if they did not map to an Ensembl Gene ID (Build 59). If a probe spanned one or more SNPs based upon Sanger Mouse Genomes sequencing [14], it was removed. Differential expression p-values were FDR adjusted using the q-value Bioconductor package [35]. Fold changes were computed using reversed VST transformations averaged for each strain.

## Results

### RNA-Seq

We generated single end RNA-Seq reads from 10 B6 and 11 D2 mice (21 lanes on three Illumina GAIIx flowcells). A high level summary of the reads for each sample is presented in **Table S1**, including total reads and uniquely mapped reads. To facilitate summarization of the RNA-Seq data relative to known gene annotations, we began by assembling a gene-level representation based upon Ensembl build 59 exon annotations as described in the Methods. This yielded summarized read counts for 36,229 genes annotated by Ensembl 59. 12,632 of these genes had no reads across all lanes and were not considered further. Another 7,414 genes had no reads for at least one B6 and one D2 sample and were considered to be unreliable. As can be seen in **Fig S1**, those genes removed tended to have very low read counts compared to the remaining genes. Our subsequent analyses focused on the remaining 16,183 genes.

**Figure 1:**
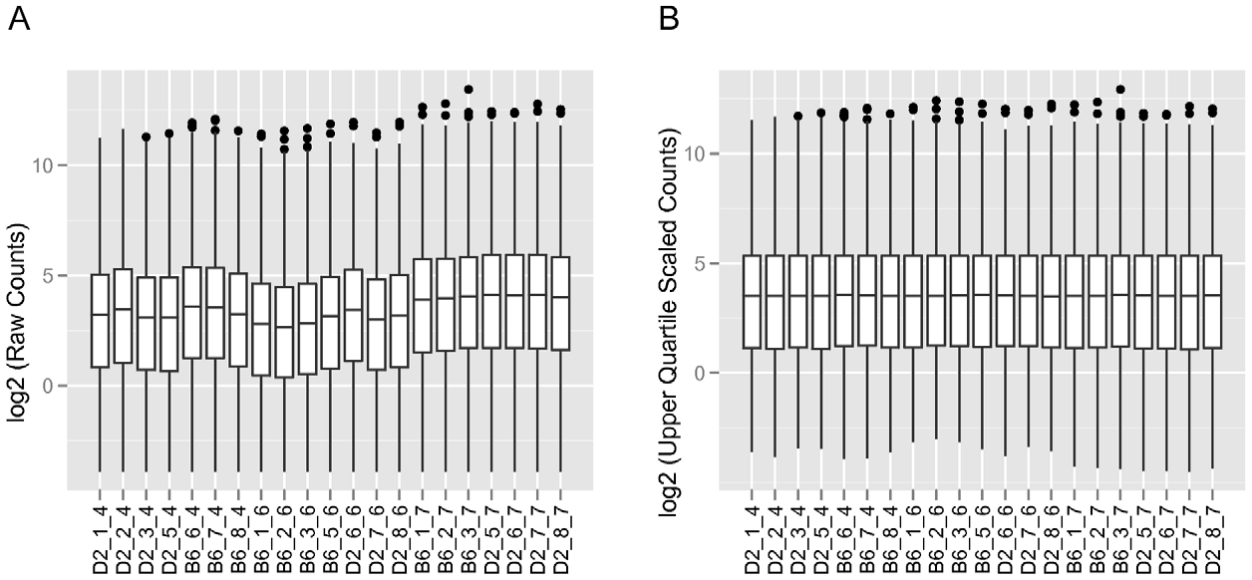
Distribution of read counts per gene per lane/flowcell. Shown in A) is the distribution of non-normalized counts per gene. The counts, however, were divided by a constant defined here as the mean of the total unique read counts in megabases (MB). This was done to facilitate comparison of the boxplots. For B), the count values were normalized by the upper quartile corrected total counts in MB. The upper quartile correction values were computed using edgeR [42]. Additionally both plots are shown on the log2 scale. Also note that the X-axis is labeled in the form strain_lane_flowcell.

Although the distribution of read counts was variable between lanes and flowcells, these differences could be normalized using an upper quartile scaling procedure [19] (**Fig. 1**) without resorting to a more extreme normalization procedure such as quantile normalization [19], [38]. Further, the upper quartile scaling (**Fig. 1**) resulted in homogenous distributions compared to those based solely on scaling by total read counts similar to the RPKM measure [15] (**Fig S2**).

In our hands, the single factor Poisson model coupled with a likelihood ratio test proposed previously [19], produced results that tended to be anti-conservative. The simplest explanation would be that there was more variability across our biological replicates than could be accounted for with a fixed dispersion Poisson model, the possibility of which has been noted before [18], [19]. Other generalized linear models could be used to account for over dispersion, such as one of a Poisson family fit with quasi-likelihood [39] or one of the negative binomial family [40]. However, because of the relative simplicity of our design, we decided to utilize an exact test [41] for the negative binomial distribution as implemented in the edgeR Bioconductor package [42].

After estimating the common dispersion parameter and applying the exact test, a false discovery rate (FDR) controlling [35] procedure was applied. We found 1,727 genes that were differentially expressed (DE) (q-value<0.01) with 958 of these being more highly expressed in B6 relative to D2 and 769 more lowly expressed in B6 relative to D2 (log2 fold change B6/D2>0 or <0, respectively). Presented in **Dataset S1** (with an accompanying description in **Text S1**) are the genes, their fold change, a measure of average read count and a flag indicating especially lowly expressed (low read count) genes. An examination of the distribution of total union exon lengths classified by whether or not the gene was determined to be differentially expressed showed that there did not seem to be appreciable gene length bias contrary to what had been seen previously [19], [43]. Examination of the counts of DE genes for each quartile of gene length also did not show any gene length bias (**Fig S3**). In contrast, if we examine the same plots generated for the single factor Poisson model incorporating the upper quartile scaling procedure read count adjustments in the offset, we see evidence that longer gene regions tend to be biased toward differential expression (**Fig S4**) indicating that choice of statistical model may be an important factor regarding the influence of gene length on differential expression.

In addition to gene length, we assessed the potential impact of genome structure on our results. As documented in Walter et al. [13], microarray analyses can detect false negative or false positive differential expression due to differences in hybridization efficiencies resulting from allelic sequence variation. A similar phenomenon could occur in RNA-Seq because of the heuristics applied to speed up realignment algorithms. For instance a combination of SNPs and base calling errors could cause a read to map erroneously or not map at all potentially causing strain-specific biases in differentially expressed genes. To address this concern we computed the number of SNPs per kilobase of the union exons for each of the 16,183 genes and assessed differential expression for evidence of directional bias. As is shown in **Fig S5** the distribution of the D2>B6 differential expression calls is shifted downward relative to the B6>D2 calls in terms of the density of known SNPs present, although the distributions largely overlap. To further address this issue we synthesized a D2 reference sequence to which we realigned the D2 reads. We found that there was a small increase in the number of uniquely mapping reads (less than 0.2% increase in reads for each D2 sample) and minimal increase in multi-mapping reads (less than 0.1% for each D2 sample). Further, after summarization, normalization, and application of the edgeR procedure, we found that 934 (97.5%) of the 958 genes originally identified as DE with B6>D2 remained DE, but status changed for the remaining 24 genes, which were no longer detected as DE indicating that they initially may have been false positives. Status also changed for 36 genes originally showing no DE; with the realigned reads, these were detected as DE with D2>B6, indicating that allele bias may have lead to false negative results. Finally, status changed for 5 genes originally detected as DE D2>B6, which were not found to be differentially expressed. Overall the number of putative false positive and false negative differential expression calls was minimal relative to the 1,727 differentially expressed genes found by RNA-Seq. The genes that changed status are indicated in **Dataset S1**.

### Microarray

The Affymetrix MOE 430 2.0 microarray contains 45,037 probesets interrogating 18,461 genes annotated in Ensembl [27], and the Illumina Mouse MouseRef-8 v2.0 microarray has 25,697 probes interrogating 16,948 genes. After analyzing each microarray experiment as described in the Methods, the Affymetrix microarray detected 10,663 genes with expression above background in the striatum, and the Illumina microarray detected 9,521 genes. Next we assessed differential expression on each microarray platform as indicated in the Methods. The Affymetrix and Illumina microarray analyses identified 1,652 and 869 differentially expressed genes (q<0.01), respectively. Inherent to the microarray platforms, there are often multiple probesets or probes mapping to the same gene. Differential expression and direction of fold change when one or more probesets or probes exist on each microarray are summarized in **Table S2** and complete details are presented in **Datasets S2** and **S3** (both described in **Text S1**). For comparing DE across platforms, we used the microarray probeset or probe with the best q-value. Presented in **Datasets S2** and **S3** are the probesets and probes, their fold change, and complete annotation details.

### Comparison of RNA-seq and microarray results

**Figure 2:**
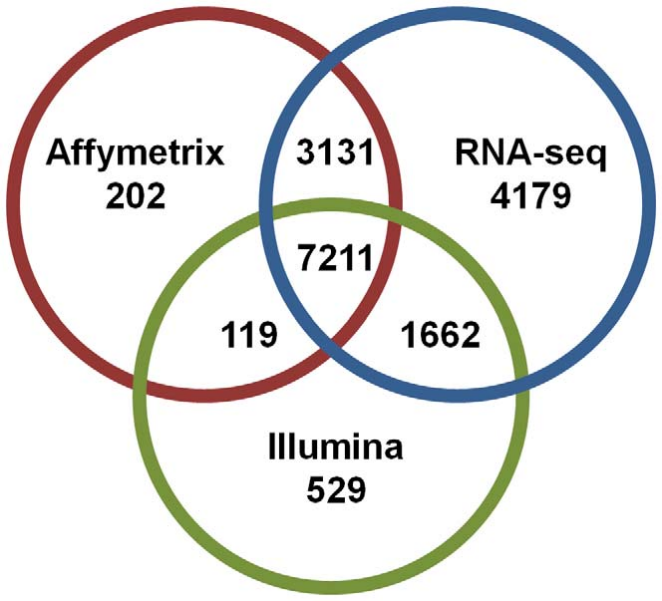
Platform overlap of detection of mouse genes expressed in striatum. For RNA-Seq: the genes that pass filters for detection when RNA-Seq data are aligned to all genes annotated in Ensembl. For microarray: the genes detected as present with at least one probeset (Affymetrix) or probe (Illumina) after filtering.

First, we compared the overlap of genes detected by each of the three platforms. 7,211 genes are common to all three platforms (**Fig. 2**). There are 202 genes unique to Affymetrix microarray, 529 unique to Illumina microarray, and 4,179 genes detected only by RNA-Seq (**Fig. 2**). The majority of these genes are protein coding, although each platform does detect some in other Ensembl gene categories as well, as annotated in the **Datasets (S1, S2, S3)**.

**Figure 3:**
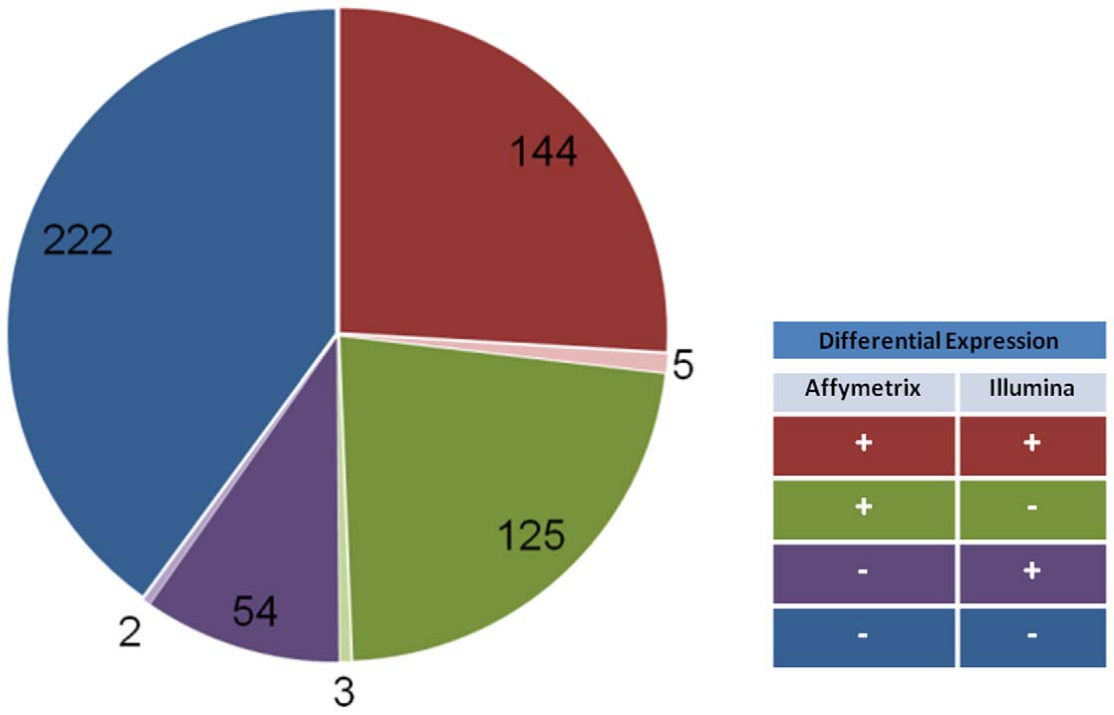
Platform overlap of differential expression (DE) detected by RNA-Seq. Of the 1524 genes DE by RNA-Seq data, 555 are also *detected* on Affymetrix MOE 430 2.0 and Illumina MouseRef-8 v2.0. When queried on all three platforms, 144 show DE on all three platforms (5 show DE on all three platforms, but do not agree in direction of fold change) indicating genes that are likely uniformly DE across all exons. 125 and 54 are DE on Affymetrix and RNA-Seq or Illumina and RNA-Seq, respectively (with 3 and 2 disagreeing in direction of fold change, respectively). While detected on all three platforms, 222 only show DE on RNA-Seq.

Next we compared the differential expression across platforms. RNA-Seq detected a total of 1,727 DE genes. The largest subset (591 genes) of the RNA-Seq DE genes were only detected as expressed in the RNA-Seq analyses, so evidence for their DE is also exclusive to the RNA-Seq data. 369 and 212 of the genes differentially expressed by RNA-Seq were detected as expressed using the Affymetrix and Illumina microarrays, but only 51% and 31% of these genes were DE in these microarray analyses, respectively. **Figure 3** summarizes the final subset of 555 RNA-Seq DE genes that are also detected as present on both microarrays by at least one probeset or probe. 222 of these genes were DE based on RNA-Seq, while both microarray platforms detected the genes as ‘present’ but did not detect DE. This suggests that differential expression may not extend across the 3′UTR, which is preferentially interrogated by the microarrays. 144 of these genes were DE using all three platforms, indicating high confidence differential expression likely across the entire gene.

Of the 555 that were compared (**Fig. 3**), only 13 were flagged as having a low read count. Further the mean average log transformed count was slightly greater in the 222 found only on RNA-Seq compared to those found differentially expressed in at least one of the array platforms (p = .015; two-tailed *t*-test) suggesting that many of the DE genes found only in the RNA-Seq analysis may indeed be true positives.

## Discussion

The present studies used a gene-level summarization approach, which has been shown to provide more reliable values than single base positions [44] or smaller intervals because of the non-uniformity observed between sequence positions that may be attributed to random hexamer priming [45]. In agreement with previous reports, we find that adjusting read count differences by the total read count seems to be inadequate, but an additional correction by the upper quartile of each lane provides a sufficient way to normalize the data [19]. We found little evidence of gene length bias using the edgeR package, though a relatively large effect was found using a Poisson model. We show that there is some potential for false positives and false negatives in comparisons of inbred mouse strains due solely to the presence of SNPs, though the effect on this comparison is not severe. We note that any bias that may exist will be a function of SNP density. For more complex and SNP dense mouse models, such as heterogenous stock [7], [46], SNPs may have a more serious impact on DE of some genes. We further note that insertions and deletions between the two strains, which we did not take into account, could potentially cause similar effects. The impact of genome structure on differential expression analysis will likely cease to be an issue for several inbred mouse strains upon release of the sequenced genomes of the common inbred strains being performed by Sanger [14], allowing each to be analyzed relative to its own reference sequence and transcript models.

RNA-Seq provided more genes that were “detected,” that is had reliable signal and were not impacted by SNP or annotation issues, than either the Illumina or Affymetrix microarray platforms. We recognize the difficulty in analytically describing a background level for RNA-Seq and for the future recommend additional experiments accurately measuring the abundance of mRNA from genes with a relatively wide range of counts in the model system of choice similar to what was done in earlier studies [15]. Despite platform differences, we found that in general, microarrays and RNA-Seq agreed relatively well based upon fold-change direction and significance values with 58% of the RNA-Seq DE genes that were interrogated on all platforms determined to be differentially expressed in the same direction at a false discovery rate of 0.01. The largest proportion of those genes that were found to be DE by RNA-Seq and at least one microarray platform were found to be DE on all three platforms. Interestingly the Affymetrix MOE430 2.0 tended to agree with the RNA-Seq data better than Illumina. Higher correlations between Affymetrix and sequencing data have been observed before in the context of the SAGE-like digital gene expression (DGE) [47].

The largest proportion of RNA-Seq DE genes interrogated by all three platforms was represented by those that were seen to be differentially expressed in RNA-Seq only. We found that these genes tended to have a greater average read count relative to those that agreed with at least one microarray platform, further indicating the utility of RNA-Seq over these two microarray platforms. The 144 genes that are differentially expressed in common with the two microarray technologies may represent instances of relatively homogenous expression across the annotated gene, as the probes from these two microarray platforms should tend to be biased towards the 3′UTR end of the gene. In this respect genes that only agreed with one platform in terms of differential expression or were only seen in RNA-Seq may represent differences in transcript isoform abundances. The variation in each of these categories illustrates an advantage of RNA-Seq compared to microarrays in that, in this case, RNA-Seq calculates DE across the entire gene rather than just at an individual probe(set) location within the gene. By some estimates there are, on average, approximately 2.5 alternative transcripts for each mouse gene [48]. With alternative splicing in the picture, probe location clearly impacts interpretation of microarray data.

Another potentially informative biological assessment of sensitivity across platforms could be done examining the Y-chromosome genes across the platforms. However, review of the Ensembl annotation utilized for the analysis in this study revealed only 20 genes annotated to be on the Y chromosome. Of these, if we examine the probes that passed our filters for SNPs, detection above background, and unique Ensembl mappings that were used in our analyses, this leaves only 7 probes on the Illumina array interrogating 5 genes on the Y chromosome and no probesets on the Affymetrix array. We do note that of the 5 genes detected by Illumina array, 4 of those were also detected by RNA-seq (the single exception was at the lowest acceptable signal level on the Illumina array). However, with so few annotated genes and such poor coverage on the arrays, assessments using this type of assay would not be informative at this time.

Additionally, many choices of experimental protocols currently exist for RNA-Seq, each with their own benefits and consequences for downstream analysis. For example to remove highly abundant rRNA molecules, either enrichment of poly-A containing sequences or depletion of the rRNAs has been used [49], [50]. For our RNA-Seq experiments we used a poly-A enrichment procedure as the current rRNA depletion strategies have been observed to be less efficient [50]. However it is possible that use of different selection procedures may impact the agreement with microarrays, which is an interesting topic for future research.

Although many methods for analyzing RNA-Seq data currently exist and more continue to be produced, the most basic and fundamental question that can be asked is whether a gene/transcript is differentially expressed between two groups. Gene level differential expression then forms the basis for further experiments directed at identifying and quantifying transcript isoforms between samples [44], [51] and *de novo* identification of unannotated expressed regions [15], [52]. Methods exist to infer alternative splicing and relative quantification of transcript isoforms [44], [51], and the generation of paired-end sequences should allow greater confidence in any novel alternative splicing events observed [53]. The use of this technology shall be pursued in the future to assess details of alternative splicing across mouse strains.

## Supporting Information

Figure S1 **Distrubution of counts from genes that were removed relative to those remaining.** The distribution of log2 mean read counts per gene categorized by whether the gene had a zero count in at least one lane for both the B6 and D2 strains (right) or either had no zero counts or zero counts in at least one lane for one of the strains (left).
(TIF)

Figure S2 **Distribution of genes scaled by the total count per lane.** Boxplots of the distribution of counts per gene scaled by the total unique read count (in megabases) for each lane.
(TIF)

Figure S3 **Relationship between gene length and gene significance for q-values computed by edgeR.** Shown in A) are the distribution of log2 gene lengths, in terms of union exon bases relative to whether that gene was determined to be differentially expressed at a q-value<.01 (DE) or not (Non DE). Shown in B) is the distribution of q-values for the gene lengths categorized by quartile of length.
(TIF)

Figure S4 **Relationship between gene length and gene significance for q-values computed using the single factor Poisson model.** Shown in A) are the distribution of log2 gene lengths, in terms of union exon bases relative to whether that gene was determined to be differentially expressed at a q-value<.01 (DE) or not (Non DE). Shown in B) is the distribution of q-values for the gene lengths categorized by quartile of length.
(TIF)

Figure S5 **Relationship between the number of SNPs per kilobase of gene length and gene differential expression direction.** The boxplot on the left (Not DE) is the distribution of SNPs per kilobase for non differentially expressed genes. The middle boxplot (DE B6>D2) shows the distribution for those genes that are both differentially expressed with the normalized count for B6 being higher than D2. Similarly the boxplot on the right shows the distribution for those genes that are differentially expressed with D2 showing a higher normalized read count than B6.
(TIF)

Table S1 **Summary of read mapping statistics.** Shown are the statistics in terms of reads for each lane of each flowcell (batch). The strain column references whether the sample was derived from the C57BL6/J (B6) or DBA2/J (D2) inbred strain of mouse. Total reads refers to the total number of generated reads for each lane. The remaining columns reference whether the read was uniquely placed to a location in the genome (Uniquely mapped), whether it mapped to more than one location (Multi-mapped) or whether it failed to map (Non-mapped). All realignments were performed using Bowtie [26].
(XLS)

Table S2 **Microarray data agreement within platform when one or more probesets or probes exist for a gene.** Numbers in table indicate number of genes. Differentially expressed (DE) at q<0.01; fold change (FC); probeset refers to both probeset for Affymetrix or probe for Illumina microarray; same direction of fold change means that the same strain showed higher gene expression; ‘some’ means at least one.
(XLS)

Text S1 Readme file for Datasets S1, S2, and S3 describing column headings.
(DOC)

Dataset S1 RNA-Seq results of all genes detected and comparisons to Affymetrix and Illumina microarray data.
(TXT)

Dataset S2 Microarray results of all probesets detected by Affymetrix MOE 430 2.0 array in B6 and D2 mouse striatum.
(TXT)

Dataset S3 Microarray results of all probesets detected by Illumina MouseRef-8 v2.0 array in B6 and D2 mouse striatum.
(TXT)
