# Supplementary figures and images for: Evaluating Gene Expression in C57BL/6J and DBA/2J Mouse Striatum Using RNA-Seq and Microarrays
Source: PLoS One. 2011 Mar 24;6(3):e17820. doi: 10.1371/journal.pone.0017820 (PMC3063777; doi:10.1371/journal.pone.0017820)

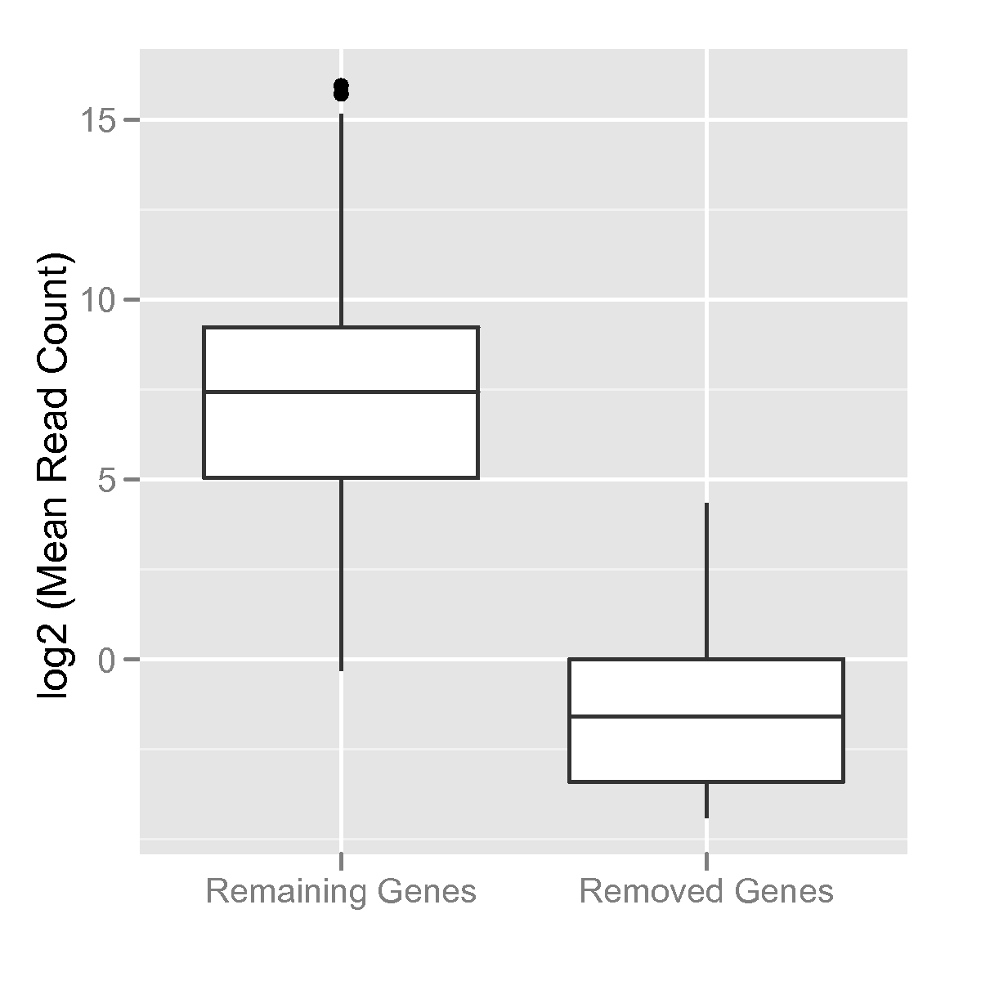

Supplement: Figure S1 — Distrubution of counts from genes that were removed relative to those remaining. The distribution of log2 mean read counts per gene categorized by whether the gene had a zero count in at least one lane for both the B6 and D2 strains (right) or either had no zero counts or zero counts in at least one lane for one of the strains (left). (TIF) [file pone.0017820.s001.tif]

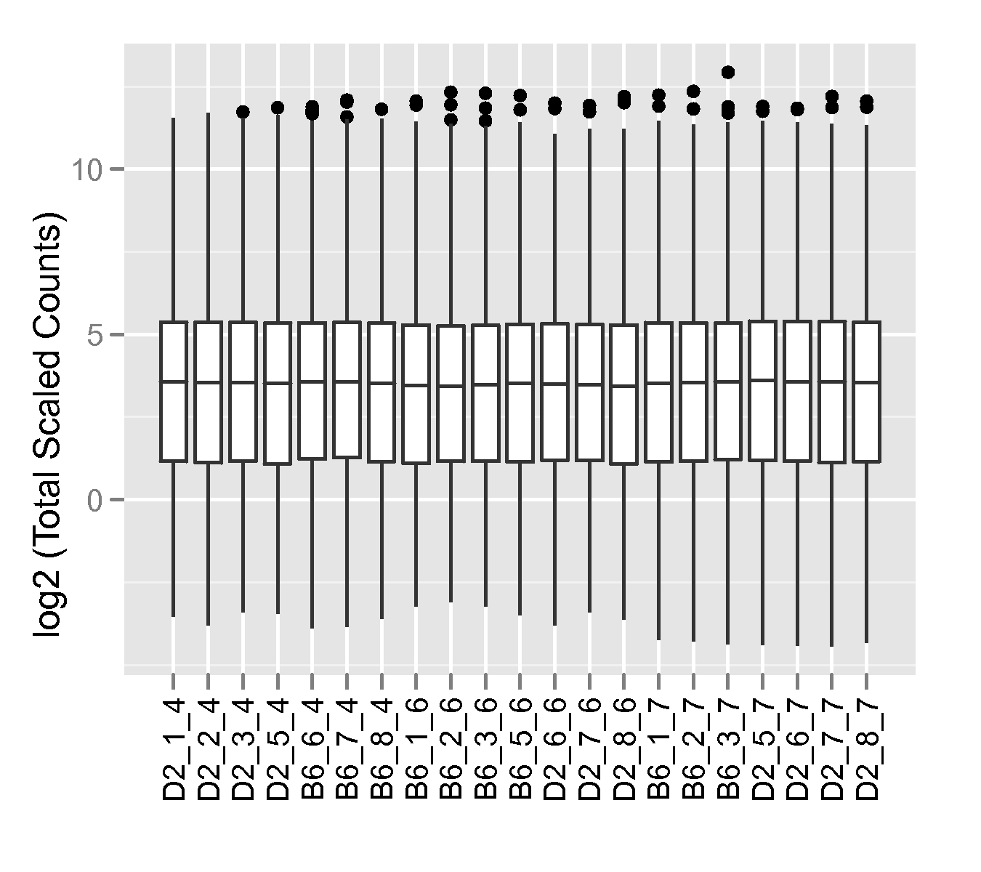

Supplement: Figure S2 — Distribution of genes scaled by the total count per lane. Boxplots of the distribution of counts per gene scaled by the total unique read count (in megabases) for each lane. (TIF) [file pone.0017820.s002.tif]

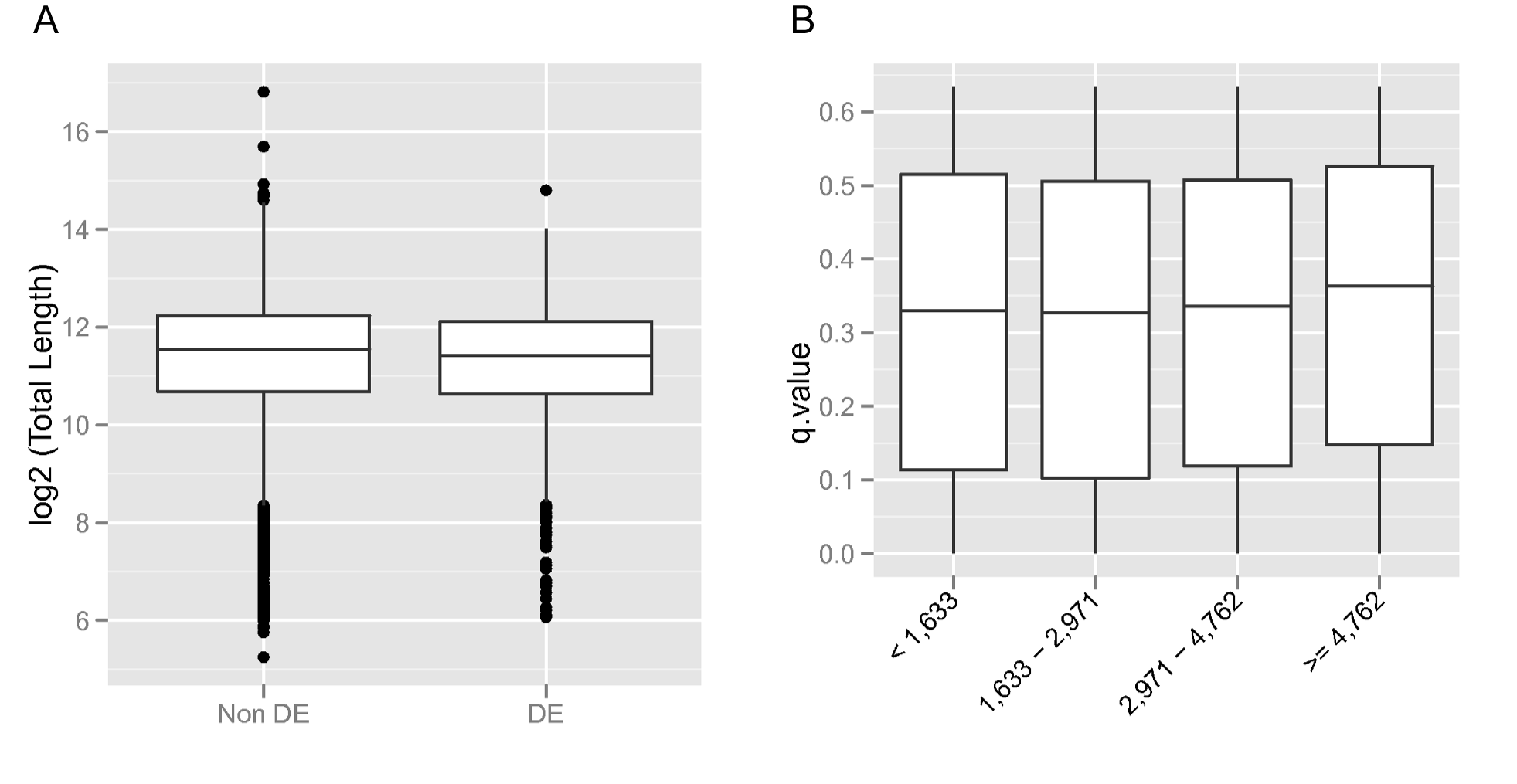

Supplement: Figure S3 — Relationship between gene length and gene significance for q-values computed by edgeR. Shown in A) are the distribution of log2 gene lengths, in terms of union exon bases relative to whether that gene was determined to be differentially expressed at a q-value<.01 (DE) or not (Non DE). Shown in B) is the distribution of q-values for the gene lengths categorized by quartile of length. (TIF) [file pone.0017820.s003.tif]

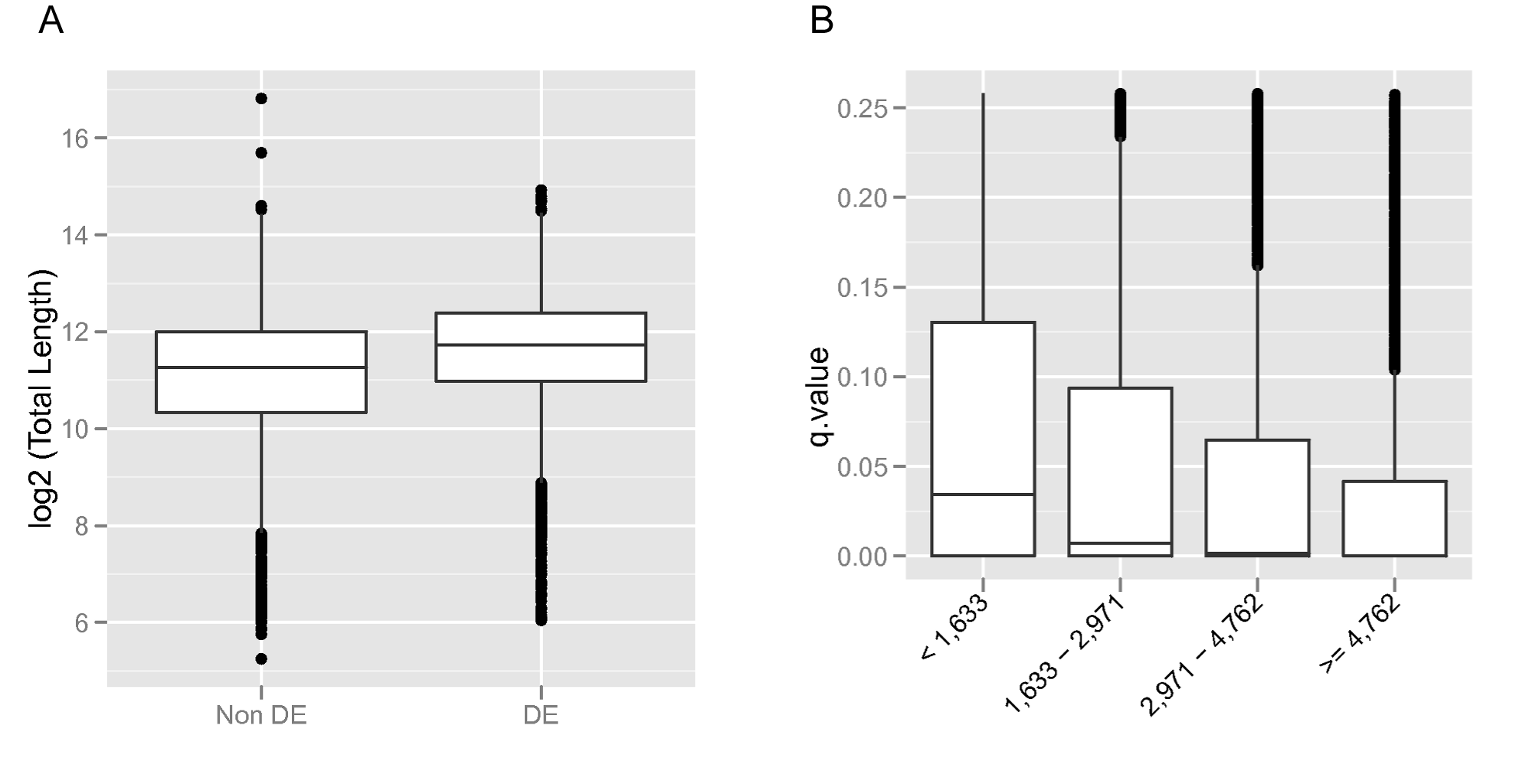

Supplement: Figure S4 — Relationship between gene length and gene significance for q-values computed using the single factor Poisson model. Shown in A) are the distribution of log2 gene lengths, in terms of union exon bases relative to whether that gene was determined to be differentially expressed at a q-value<.01 (DE) or not (Non DE). Shown in B) is the distribution of q-values for the gene lengths categorized by quartile of length. (TIF) [file pone.0017820.s004.tif]

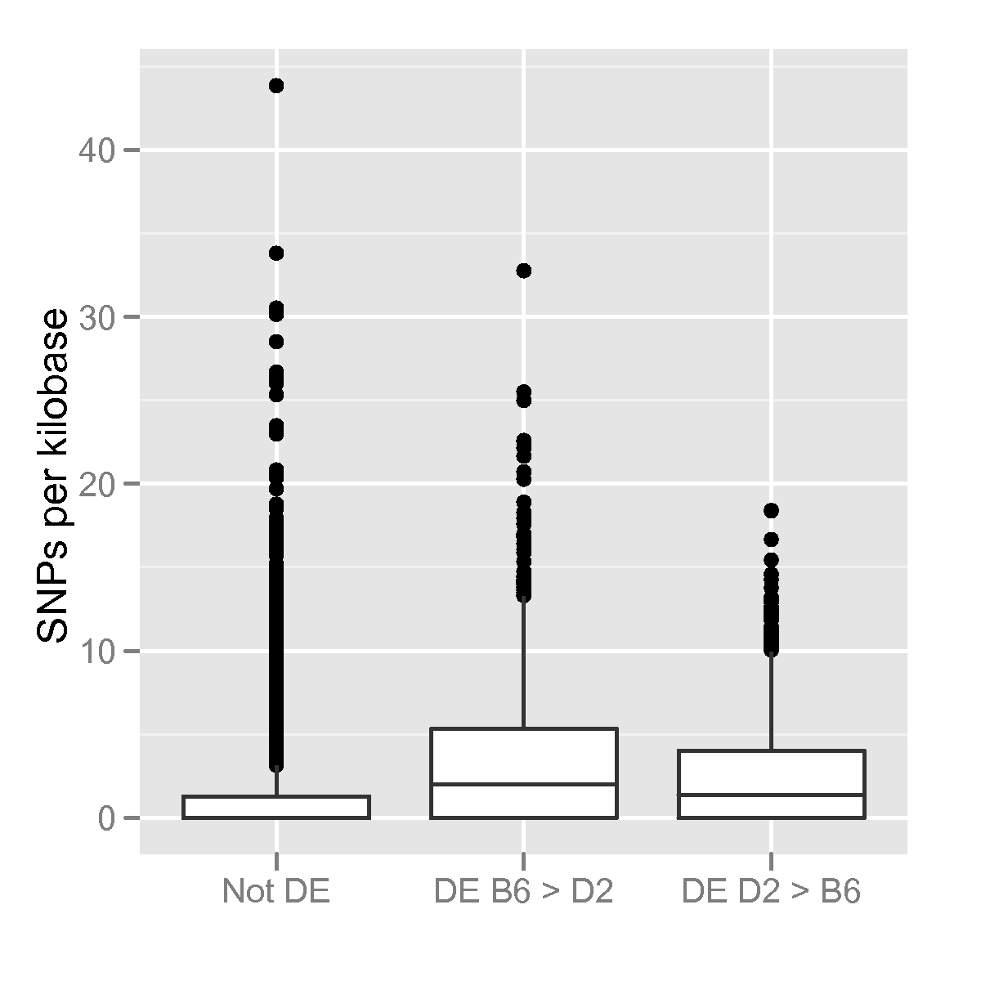

Supplement: Figure S5 — Relationship between the number of SNPs per kilobase of gene length and gene differential expression direction. The boxplot on the left (Not DE) is the distribution of SNPs per kilobase for non differentially expressed genes. The middle boxplot (DE B6>D2) shows the distribution for those genes that are both differentially expressed with the normalized count for B6 being higher than D2. Similarly the boxplot on the right shows the distribution for those genes that are differentially expressed with D2 showing a higher normalized read count than B6. (TIF) [file pone.0017820.s005.tif]
