# Supplementary material for: Evaluating Gene Expression in C57BL/6J and DBA/2J Mouse Striatum Using RNA-Seq and Microarrays
Source: PLoS One. 2011 Mar 24;6(3):e17820. doi: 10.1371/journal.pone.0017820 (PMC3063777; doi:10.1371/journal.pone.0017820)
Supplement: Text S1 — Readme file for Datasets S1, S2, and S3 describing column headings. (DOC) [file pone.0017820.s008.doc]

README for Datasets S1, S2, and S3

Dataset_s1.txt

This file contains Illumina GAIIx RNA-Seq analyses.

n=10 striatum C57BL/6J males

n=11 straitum DBA/2J males

16,183 genes with read counts above background

Column headings and explanations:

Ensembl

Gene ID from Ensembl.org

Gene_Name

logConc

edgeR averaged across all 21 samples

p.value

Fold_Change

B6/D2: antilog (base 2) of average B6/average D2

q.value

FDR

DE

Differential Expression

1 q < 0.01

0 q > 0.01

Low_read_count

1 logConc < -20

0 logConc >= -20

D2_SNP_correction

1 DE B6 > D2 STD; non DE SNPCOR -- Upregulated in B6 in the original analysis but became non differentially expressed after D2 SNP correction.

2 DE D2 > B6 STD; non DE SNPCOR -- Upregulated in D2 in the original analysis but became non differentially expressed after D2 SNP correction.

3 In STD; Not in SNPCOR -- The gene that was in the original analysis but was excluded (because of zeros) from the second analysis.

4 No change -- No change in status between the original and D2 SNP corrected analyses.

5 non DE STD; DE B6 > D2 SNPCOR -- Non differentially expressed in the original but became upregulated in B6 after D2 SNP correction.

6 non DE STD; DE D2 > B6 SNPCOR -- Non differentially expressed in the original but became upregulated in D2 after D2 SNP correction.

Affymetrix_ProbeID

Affymetrix probeset with the lowest q value that was used in cross platform comparisons

flip_flop

If RNA-seq showed DE, then

1 Affymetrix and/or Illumina DE disagreed on direction of fold change

0 Affymetrix and/or Illumina DE confirmed direction of fold change

Affymetrix_Fold_Change

B6/D2: antilog (base 2) of the RMA values of average B6/average D2

Affymetrix_q.value

FDR

Illumina_Probe_ID

Illumina probe with the lowest q value that was used in cross platform comparisons

Illumina_Fold_Change

B6/D2: inverse VST values of average B6/average D2

Illumina_q.value

FDR

Biotype

Gene Biotype from Ensembl

Chr

Chromosome

Gene_start

basepairs

Gene_Description

from Ensembl

Dataset_S2.txt

This file contains Affymetrix MOE430 2.0 microarray analyses.

n=10 striatum C57BL/6J males and females

n=10 straitum DBA/2J males and females

This data has been sex-corrected. See Methods in manuscript for details.

15,814 probesets that pass filters

Column headings and explanations:

Ensembl

Gene ID from Ensembl.org

Gene_Name

ProbeID

Affymetrix MOE430 2.0 Probe ID

Average_RMA

average RMA value across all 20 samples

p.value

p value of RMA

Fold_Change

B6/D2: antilog (base 2) of the RMA values of average B6/average D2

q.value

FDR

DE

Differential Expression

1 q < 0.01

0 q > 0.01

Probes_per_gene

Total number of Affymetrix probes that pass filters for each gene

Multiple_probes

For the 1945 probes that are differentially expressed (q<0.01), the following codes indicate results for the other probes for the same gene

1 All probesets for this gene are DE and all in same direction fold change (dir FC);

2 Only one probeset for this gene and it is DE;

3 All probesets for this gene are DE but flip flop dir FC;

4 Probesets for this gene DE and not DE;

5 Probesets for this gene DE but flip flop dir FC and at least one probeset not DE

Biotype

Gene Biotype from Ensembl

Chr

Chromosome

Gene_start

basepairs

Gene_Description

from Ensembl

Dataset_S3.txt

This file contains Illumina MouseRef-8 v2.0 microarray analyses.

n=12 striatum C57BL/6J males

n=12 straitum DBA/2J males

12,722 probes that pass filters

Column headings and explanations:

Ensembl

Gene ID from Ensembl.org

Gene_Name

Probe_ID

Illumina MouseRef-8 v2.0 Probe_ID

nuID

Average_lumi

average lumi value across all 24 samples

p.value

p value of lumi

Fold_Change

B6/D2: inverse VST values of average B6/average D2

q.value

FDR

DE

Differential Expression

1 means q < 0.01

0 means q > 0.01

Probes_per_gene

Total number of Illumina probes that pass filters for each gene

Multiple_probes

For the 943 probes that are differentially expressed (q<0.01), the following codes indicate results for other probes for the same gene

1 All probes for this gene are DE and all in same direction fold change (dir FC);

2 Only one probe for this gene and it is DE;

3 All probes for this gene are DE but flip flop dir FC;

4 Probes for this gene DE and not DE;

5 Probes for this gene DE but flip flop dir FC and at least one probeset not DE

Biotype

Gene Biotype from Ensembl

Chr

Chromosome

Gene_start

basepairs

Gene_Description

from Ensembl
